# Histoplasmosis Associated With Bat Guano Exposure in Cannabis Growers: 2 Cases

**DOI:** 10.1093/ofid/ofae711

**Published:** 2024-12-05

**Authors:** Paulina Sudnik, Patrick Passarelli, Angela Branche, Ellen Giampoli, Ted Louie

**Affiliations:** Department of Infectious Diseases, University of Rochester, Rochester, New York, USA; Department of Infectious Diseases, University of Rochester, Rochester, New York, USA; Associate Professor of Medicine, Department of Infectious Disease at the University of Rochester, Rochester, New York, USA; Associate Professor Departments of Pathology and Laboratory Medicine and Otolaryngology at the University of Rochester, Rochester, New York, USA; Associate Professor of Medicine, Department of Infectious Disease at the University of Rochester, Rochester, New York, USA

**Keywords:** bat guano, cannabis, cultivation, fertilizer, *histoplasma capsulatum*

## Abstract

*Histoplasma capsulatum* is a pathogenic dimorphic fungus with evolving epidemiology. Initially described as endemic to the Ohio and Mississippi river valleys, the infection now regularly occurs in central and eastern United States, with cases reported across the entire country. Transmission happens via inhalation of conidia during activities that disturb fungal hyphae. Sporadic cases related to individual exposures now outnumber work-related outbreak cases in the United States. We describe 2 fatal cases of histoplasmosis in Rochester, New York, associated with exposure to bat guano as a fertilizer for cannabis cultivation, including 1 case in which it was commercially obtained.

## CASE 1

**Figure 1.**
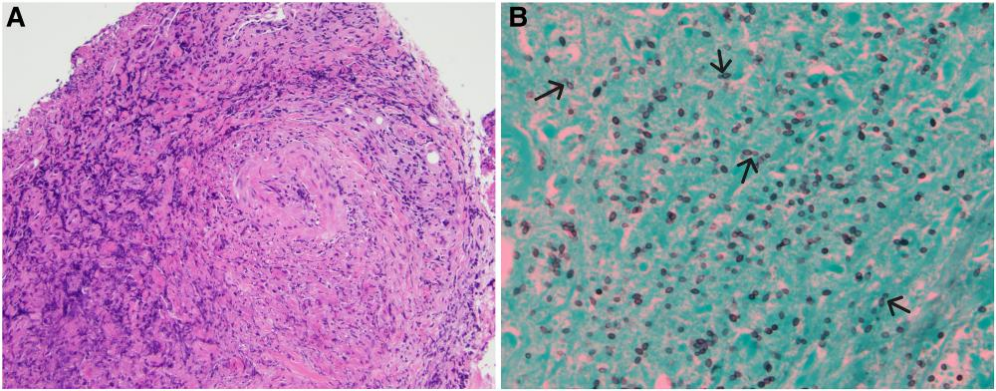
Supraglottic biopsy. (*A*) The image shows ulceration with loss of surface epithelium. Beneath is marked acute and chronic inflammation with necrotic cellular debris throughout the biopsy. Perivascular inflammation is prominent. No definitive granulomas are identified. The absence of granuloma formation may be attributed to the patient's use of a tumor necrosis factor-alfa inhibitor (hematoxylin and eosin, 10×). (*B*) Gomori-methanamine silver stain highlights abundant small oval yeast forms with occasional narrow-based budding. No pseudohyphae are identified. The histologic differential was narrowed to histoplasmosis, cryptococcosis, and emergomycosis. The mucicarmine stain was negative, which ruled out classic Cryptococcus. The clinical picture was not consistent with emergomycosis. ©2024 Ellen Giampoli

A 59-year-old man with emphysema, heavy tobacco and inhalational cannabis use, rheumatoid arthritis on adalimumab, and recent imaging suspicious for a laryngeal mass was admitted to Strong Memorial Hospital for respiratory failure. He had a sore throat, progressive dysphagia, and weight loss for at least 6 weeks before the presentation. An outpatient computed tomography (CT) scan of the neck revealed irregular circumferential mucosal prominence at the level of the supraglottic larynx and the right proximal vocal cord. On admission, the patient appeared cachectic and was septic. He was found to have *Streptococcus pneumoniae* bacteremia. A CT of the chest showed widespread multilobar consolidations, superimposed on a background of severe apical emphysema and bilateral tree-in-bud nodularity. He was treated for pneumonia and bacteremia with piperacillin-tazobactam with improvement in fever, but still required variable oxygen supplementation and invasive ventilation for a brief period. He underwent direct laryngoscopy that revealed vestibular fold thickening and irregularity of the mucosa. Carcinoma was suspected and biopsy was obtained without cultures. The biopsy showed yeast forms morphologically consistent with *Histoplasma* (Figure 1*A*, 1*B*). On exposure assessment, he reported the use of bat guano from an online store as a fertilizer for cannabis plants that he cultivated and smoked, but he denied other potential exposures. He was started on amphotericin B for suspected pulmonary and presumed laryngeal histoplasmosis. His *Histoplasma* urine antigen level was 11.12 ng/mL (cutoff >0.2 ng/mL) and beta-d-glucan 312 pg/mL (cutoff >80 pg/mL). Sputum culture and cultures from repeat pharyngeal biopsy grew *Histoplasma capsulatum*. The patient was treated with 2 weeks of liposomal amphotericin B followed by itraconazole for presumed disseminated histoplasmosis. *Histoplasma* urine antigen trended down to 6.34 ng/mL after 2 weeks of treatment. The patient had recurrent aspiration events complicated by respiratory failure; he ultimately transitioned to comfort care and died.

## CASE 2

A 64-year-old man with severe peripheral arterial disease and prior aorto-bifemoral bypass surgery, tobacco, inhalational cannabis use, and alcohol use was hospitalized for hypo-osmolar hyponatremia, poor oral intake associated with poor dentition, and a 35-pound weight loss over several months. He had a chronic cough with scarce sputum production. Remarkably, the patient reported a heavy bat infestation of his attic with a thick layer of guano that he inspected on multiple occasions intending to use it as fertilizer for his cannabis plants. He denied other potential exposures to *Histoplasma*. On admission, he had a fever of 38 °C and his body mass index was 14.8 kg/m^2^. A CT of the chest revealed multiple bilateral thin and thick-walled cavitary lesions. A CT of the abdomen showed numerous pancreatic cystic lesions, extensive soft tissue thickening, and cystic lesions in the retroperitoneum extending into bilateral adrenal glands, with the largest peripancreatic lesion measuring 8.8 × 5.6 cm. Infectious workup was notable for *Histoplasma* urine antigen of 11.56 ng/mL, and beta-D-glucan >500 pg/mL. Biopsy of the pancreatic lesion was offered to rule out malignancy, but the patient declined. He was treated with itraconazole for suspected pulmonary, pancreatic, and adrenal histoplasmosis. An interval CT of the abdomen 3 weeks after presentation and 2 weeks into therapy demonstrated a significant decrease in the size of the pancreatic and retroperitoneal lesions; the largest peripancreatic cystic lesion decreased to 2.0 × 1.4 cm. The patient was discharged but readmitted to the hospital 1 month after with abdominal pain. A CT scan of the chest showed a mixed response in the pulmonary cavitary lesions, and a CT of the abdomen demonstrated near-complete resolution of retroperitoneal lesions, diffuse colonic pneumatosis, and portal mesenteric venous gas. The patient ultimately died of complications related to bowel ischemia.

## DISCUSSION

In the United States, original histoplasmosis-endemic areas were established in the 1940s and 1950s through nationwide sensitivity skin testing, which revealed sensitization rates of 60%–90% in the populations of the Ohio and Mississippi River valleys [1]. Recent literature suggests that *Histoplasma* infections are more geographically widespread in the United States than previously described [2, 3]. The majority cases are sporadic rather than part of outbreaks [4]. Multiple factors have influenced the distribution of cases, including improved diagnostic tests, the use of protective equipment in workplaces, population migration, climate change, and increased proportion of immunocompromised population [5]. Behavioral factors also contribute to the disease epidemiology [6].

The association between histoplasmosis cases and outbreaks with environmental disturbances, particularly in the presence of bat and bird droppings, is well recognized [7]. Bats are one of the few species infected by *H capsulatum* [8]. Bird and bat droppings provide an ideal medium for *Histoplasma* growth and serve as a source of the fungus in the environment. Notably, using bat guano or bird droppings as fertilizer may contribute to the infection with *H capsulatum*. Outbreaks of histoplasmosis related to agricultural fertilizers have been detected in Latin America [9, 10]. Remarkably, numerous media articles promote bat guano as a “natural superfood” for cannabis plants because of its richness in nitrogen, phosphorus, potassium, and organic compounds.

These cases emphasize 2 potential sources of exposure: commercial fertilizers and natural bat guano. Neither federal governmental agencies nor the New York State Department of Agriculture appear to routinely test commercial bat guano for *H capsulatum* [11–13]. The fungus is difficult to culture from a polymicrobial environmental source, and there are limited molecular methods for testing [14]. Therefore, it is important to develop strategies for testing biofertilizers containing bat guano before they reach the market. If the testing is not feasible, risk mitigation strategies should be implemented, such as labeling the product with a warning sign and providing instructions on safe use. Cannabis growers can collect natural guano in caves or other bat habitats. The tolerance of bats in proximity to humans, handling of guano, and exploration of bat-infested areas may significantly impact the epidemiology of the disease. Given the recent legalization [15, 16] and an expected increase in home cultivation [17] of cannabis, along with the promotion of bat guano for this purpose, it is important to raise public awareness about the potential risk of using bat guano as fertilizer and emphasize the need for protective measures, such as wearing masks when handling it.

These cases also highlight that growing cannabis can be a relevant part of a patient's history as a risk factor for histoplasmosis. A wide range of the population may be at risk of acquiring the disease through this exposure, with the highest risk among immunocompromised individuals, particularly those with advanced HIV infection, patients treated with tumor necrosis factor inhibitors, and organ transplant patients [18]. Clinicians need to ask about bat guano exposure in cannabis growers to discuss preventive measures and establish timely diagnoses.

## CONCLUSION

Exposure to bat guano among cannabis growers appears to be a recent trend that can lead to histoplasmosis cases and outbreaks. It is crucial to raise awareness among physicians and patients to reinforce personal preventive measures and establish timely diagnosis. Commercial biofertilizers containing bat guano should be tested for *H capsulatum* before reaching the market. If testing is not feasible, risk mitigation strategies should be implemented.

## Notes

***Acknowledgment*.** The authors acknowledge Paul Graman for his contribution to the epidemiologic perspective. Copilot AI Software was used for proofreading of the text.

***Patient consent statement*.** The patients described in the case report unfortunately have deceased and we cannot obtain a written consent form the patients.

## References

[1] Manos NE, Ferebee SH, Kerschbaum WF. Geographic variation in the prevalence of histoplasmin sensitivity. Dis Chest 1956; 29:649–68. 13317782 10.1378/chest.29.6.649

[2] Ashraf N, Kubat RC, Poplin V, et al Re-drawing the maps for endemic mycoses. Mycopathologia 2020; 185:843–65. 32040709 10.1007/s11046-020-00431-2 PMC7416457

[3] "Areas with Histoplasmosis" U.S. Centers for Disease Control and Prevention, April 24, 2024, accessed November 2024, Available at: https://www.cdc.gov/histoplasmosis/data-research/maps

[4] Benedict K, Mody RK. Epidemiology of histoplasmosis outbreaks, United States, 1938–2013. Emerg Infect Dis 2016; 22(3)):370–8. 26890817 10.3201/eid2203.151117 PMC4766901

[5] de Perio MA, Benedict K, Williams SL, et al Occupational histoplasmosis: epidemiology and prevention measures. J Fungi (Basel) 2021; 7:510. 34206791 10.3390/jof7070510 PMC8306883

[6] Deepe GS Jr . Outbreaks of histoplasmosis: the spores set sail. PLoS Pathog. 2018; 14:e1007213. 30212569 10.1371/journal.ppat.1007213 PMC6136805

[7] Benedict K, Toda M, Jackson BR. Revising conventional wisdom about histoplasmosis in the United States. Open Forum Infect Dis 2021; 8: ofab306. 34703835 10.1093/ofid/ofab306 PMC8538056

[8] Rodrigues AM, Beale MA, Hagen F, et al The global epidemiology of emerging Histoplasma species in recent years. Stud Mycol 2020; 97:100095. 33335607 10.1016/j.simyco.2020.02.001 PMC7714791

[9] Taylor ML, Ruíz-Palacios GM, del Rocío Reyes-Montes M , et al Identification of the infectious source of an unusual outbreak of histoplasmosis, in a hotel in Acapulco, state of Guerrero, Mexico. FEMS Immunol Med Microbiol 2005; 45:435–41. 10.1016/j.femsim.2005.05.017 16061362

[10] Jiménez RA, Urán ME, de Bedout C, et al Brote de histoplasmosis aguda en un grupo familiar: identificación de la fuente de infección [outbreak of acute histoplasmosis in a family group: identification of the infection source]. Biomedica 2002; 22:155–9. 12152481

[11] McCombie W, Bryant-Spas S. "Five ways to ease the regulatory journey for biological products" TSG Consulting, April 16, 2024, accessed November 2024, Available at: https://www.tsgconsulting.com/news-detail/regulatory-journey-biological-products

[12] "Agriculture Nutrient management and Fertilizer" US EPA, January 25, 2024, accessed November 2024, Available at: https://www.epa.gov/agriculture/agriculture-nutrient-management-and-fertilizer

[13] "Commodities Program: Fertilizer; Plant and Soil Inoculants" New York State Department of Agriculture and Markets, accessed November 2024, Available at: https://agriculture.ny.gov/plant-industry/commodities-program

[14] Gómez LF, Torres IP, Jiménez-A MDP, et al Detection of histoplasma capsulatum in organic fertilizers by Hc100 nested polymerase chain reaction and its correlation with the physicochemical and microbiological characteristics of the samples. Am J Trop Med Hyg 2018; 98:1303–12. 29532772 10.4269/ajtmh.17-0214 PMC5953352

[15] “Where Is Marijuana Legal in the US? A State-by-State Guide.” NBC, 2024, accessed 8/1/2024, Available at: https://news.gallup.com/poll/284135/percentage-americans-smoke-marijuana.aspx

[16] "What Percentage of Americans Smoke Marijuana?" Gallup, November 1, 2024, accessed November 2024, Available at: https://news.gallup.com/poll/284135/percentage-americans-smoke-marijuana.aspx

[17] Wadsworth E, Schauer GL, Hammond D. Home cannabis cultivation in the United States and differences by state-level policy, 2019–2020. Am J Drug Alcohol Abuse 2022; 48(6)):701–11.. 36288408 10.1080/00952990.2022.2132507

[18] Azar MM, Loyd JL, Relich RF, Wheat LJ, Hage CA. Current concepts in the epidemiology, diagnosis, and management of histoplasmosis syndromes. Semin Respir Crit Care Med 2020; 41(1)):13–30. 32000281 10.1055/s-0039-1698429

